# Historical and future projected costs of capital for ten energy technologies across 176 countries

**DOI:** 10.1038/s41597-025-06177-0

**Published:** 2025-12-04

**Authors:** Luke Hatton, Iain Staffell, Malte Jansen, Gbemi Oluleye, Adam Hawkes

**Affiliations:** 1 Department of Chemical Engineering, Imperial College London, Exhibition Rd, London, SW7 2AZ UK; 2 Centre for Environmental Policy, Imperial College London, Exhibition Rd, London, SW7 2AZ UK; 3 Science Policy Research Unit, University of Sussex, Brighton, BN1 9RH UK

**Keywords:** Energy economics, Wind energy, Energy modelling, Solar energy, Renewable energy

## Abstract

Accelerating the deployment of clean generation technologies will be key to achieving global climate targets, yet accurately modelling the financial conditions they face is challenging. The cost of capital (or discount rate) is a key input for energy system models, which are used widely to explore future decarbonisation scenarios. Despite its importance, data on the cost of capital is typically outdated, closed-source and geographically concentrated. Even for countries with substantial technology deployment, accessing empirical data can be difficult, leading to the use of standard assumptions in modelling which can substantially bias results (due to the high capital intensity of clean technologies). Here, we provide estimates of the cost of capital for 10 generation technologies at a national level (including solar, wind, bioenergy, and natural gas with carbon capture) for 176 countries, for 2015 to 2030 spanning 27,640 data points. An interactive web tool available at *wacc-forecaster.streamlit.app* has also been produced to visualise estimates for given years, countries and technologies, making results more accessible to a range of audiences.

## Background & Summary

Meeting global climate goals will require the rapid transition away from fossil fuels to an energy system based on low-carbon technologies, principally renewables^1^. To deliver emissions reductions at the required pace, both research and deployment of emerging technologies such as hydrogen and carbon capture and storage will also need to scale^2^, particularly in industrialising countries such as China^3,4^. A wide range of tools are used by stakeholders in industry, academia and government to develop insights to inform business decisions and aid policy design within the energy transition, including energy system models (ESMs)^5^. A range of factors, including future cost uncertainties^6^, societal factors^7^ and difficulties depicting developmental contexts^8–10^ pose challenges to the accuracy of technical insights from ESMs^11^. Cost scenarios are also often linked to technology learning curves, with the strong bearing of initial technoeconomic assumptions on future cost scenarios necessitating effective treatment of uncertainties^12^. For developing countries in particular, limited availability of empirical data to guide input assumptions and support model calibration can pose substantial challenges^8,13^. Given that future emissions trajectories will depend heavily on whether development in low- and middle-income countries locks in fossil infrastructure^14^ and follows the carbon-intensive trajectory of high-income countries^15^, ensuring accurate technical insights to aid energy policymaking in developing countries will be key to global climate goals^10^.

**Fig. 1:**
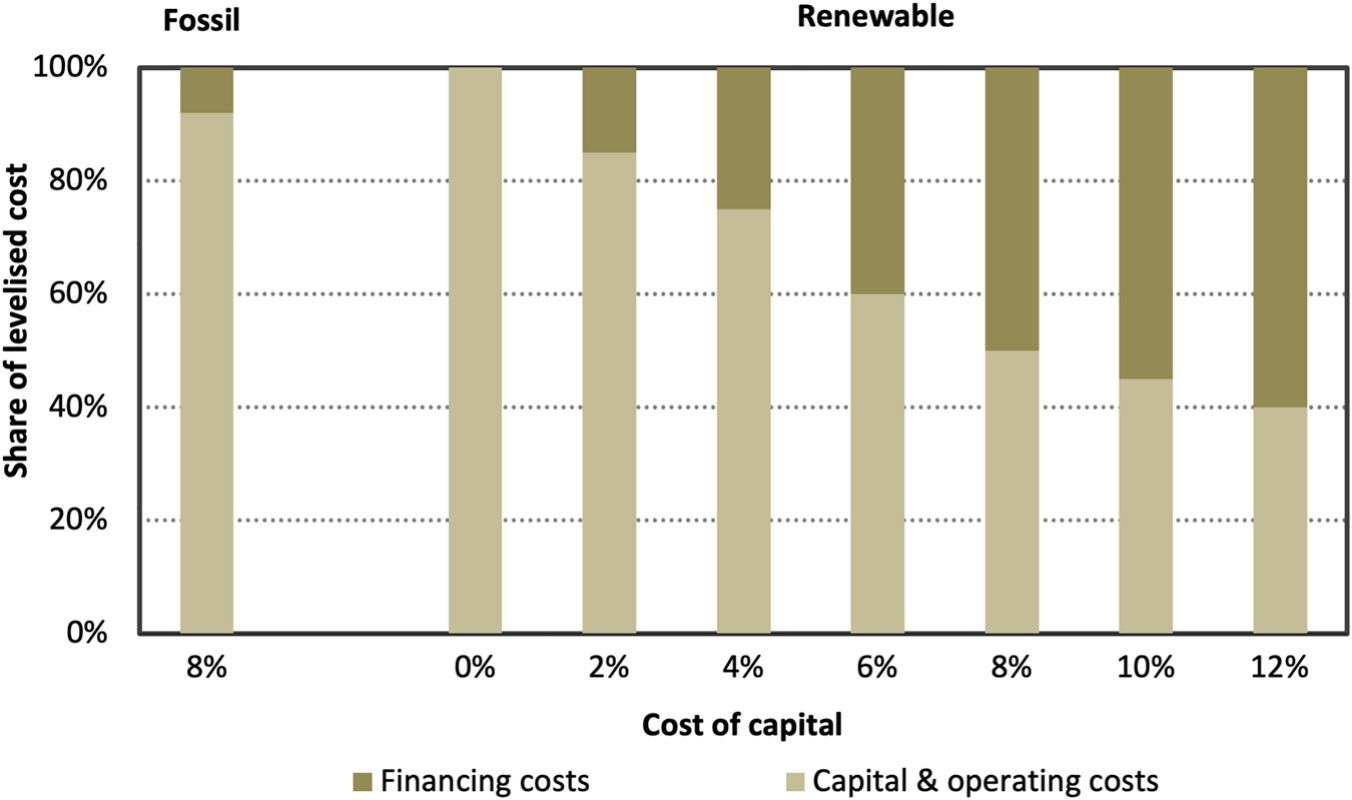
The share of the total levelised cost of electricity (LCOE) attributable to financing costs compared to capital & operating costs, compared between renewables and fossil technologies. Data on the breakdown of contributions of financing, operating and capital costs to the LCOE is taken from Bullard^66^.

Existing research has highlighted the importance of correct assumptions for the cost of capital (the financing terms for a given investment) to the accuracy and utility of technical insights derived from ESMs^16–19^. For clean technologies, which typically constitute high upfront investments with low ongoing operating costs, electricity costs and project viability are highly sensitive to the cost of capital^20,21^. Financing costs make up around 50% of the lifetime costs for renewable projects at a cost of capital of 8%, more than five times greater than the share for fossil-fuelled electricity generation as highlighted by Fig. 1. The cost of capital substantially affects this share and so can push up the levelised cost of electricity for renewables substantially. In developing countries, the cost of capital can be up to three times higher than comparable projects in high-income nations^22^, up to a reported maximum of 18%^23^, meaning that the share of the LCOE corresponding to financing costs may be even higher for some projects and geographies. Despite its importance, empirical data on financing terms are typically regarded as trade secrets, while openly-available data are typically difficult to access, geographically limited and/or out of date^24^ – even in high-income contexts.

High costs of capital in developing countries pose a challenge to the pace, equity and affordability of the energy transition^25^. Effective design of de-risking programmes^26^, suitable policy support^27,28^ and a scale-up concessional financing^29,30^ are important to addressing these issues. In the case of the latter, volumes will not only need to be scaled but support will need to be strategically directed to maximise mobilisation of private capital^31^. Lack of data or uncertainty on commercial investment terms also poses a challenge to setting sufficiently balanced concessional rates^32^ and increases the difficulty in assessing the cost-effectiveness of interventions (alongside limited reporting and project-level evaluations^33^). Understanding the likely cost of capital for energy-related investments will therefore be key to defining cost-effective support and ensuring that technical insights from energy system models are as relevant for policymakers and international stakeholders as possible^12,19^.

Several methodologies have been developed to estimate or elicit the cost of capital for energy technologies^24^, including through expert interviews^34,35^, back-calculation of auction results^36–38^ and estimates of the cost of debt and equity^34,39–41^. A full discussion of the approaches used for estimating the cost of capital can be found in Steffen 2020^24^, which systematically reviewed the methods used to estimate the cost of capital and found four general approaches: elicitation of project finance data, survey of experts, replication of auction results and analysis of financial market data. Many of the existing applications of these approaches are limited in geographic scope and temporal range, due to data availability challenges^24^ and the geographic concentration of existing renewables deployment. Due to the difficulties in accessing empirical data, verification of estimates or stakeholder-informed ranges is also tough. Reported empirical values of the cost of capital can also show substantial variation within individual markets^23,24^ and across technologies^35^, due to the strong impact of differences at the project and investor level on the overall cost of capital^35^.

Financing terms for clean generation technologies also vary over time in addition to between countries and across technologies, due to fluctuations in national interest rates^42^, increased financing experience with a given technology^18,43^, and changes in national and global macroeconomic environments^44^. For project developers and nationalpolicymakers, understanding how short-term changes are likely to affect the cost of capital is valuable, despite the uncertainty inherent in projections of economic parameters, but this has received limited examination to date. For example, the International Energy Agency assumes a standardised range of the cost of capital of 4–9% in real terms for clean generation technologies in its long-term modelling^45^, unlike its approach to deployment costs which includes regionally distinct projections under a range of different deployment scenarios. Whilst it is challenging to project likely changes in the cost of capital beyond the short term, scenario analysis can and should be used to explore potential cost of capital scenarios and how these are likely to impact on the future economic viability of clean generation technologies^41,46^.

Here, we construct a model to evaluate the cost of debt, equity and overall cost of capital for 176 countries, covering 10 key electricity generation technologies selected because of a) their importance to global power sector decarbonisation and b) the availability of existing data. The model covers the following technologies: 1) solar photovoltaic (hereon PV), 2) onshore wind, 3) offshore wind, 4) hydroelectric power, 5) biomass, 6) natural gas combined cycle turbines (CCGTs), 7) CCGTs equipped with carbon capture and storage technologies (CCS), 8) geothermal, 9) tidal and 10) wave power. Nuclear power and emerging technologies such as green hydrogen were not included because of differences in their risk profiles, though future work should explore their financing conditions in detail. We integrate financial market parameters and data on electricity generation to provide country-level estimates at a yearly resolution. Building on approaches from other studies, we also provide short-term projections of the cost of capital out to 2030 based on electricity market and macroeconomic trajectories, which are also key inputs to ESMs, spanning a total of 27,640 data points^47^. The methodology presented here allows for easy modification of the underlying assumptions, with access to the model also provided through an interactive web tool published at *wacc-forecaster.streamlit.app*.

**Fig. 2:**
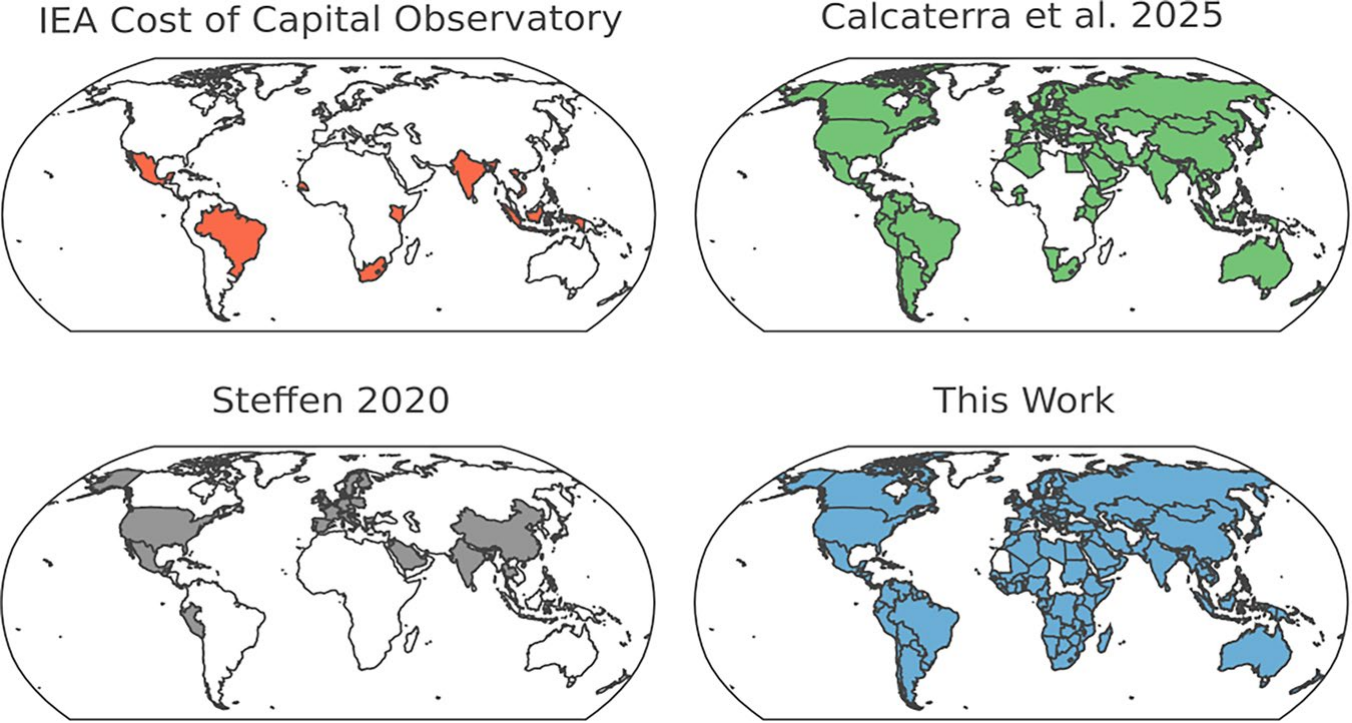
Geographic coverage of the estimates presented in this work, compared to selected works that estimate and/or collate cost of capital data for multiple generation technologies, i.e. Calcaterra *et al*.^41^, Steffen^24^, and IEA^23^. Note that IRENA’s estimates^34^ cover the same countries as Calcaterra *et al*.^41^.

Figure 2 plots the range of countries covered by our model, with the geographic coverage of three other studies which collate or estimate the cost of capital for multiple technologies highlighted alongside it. Our estimates cover a total of 176 countries, with increased coverage of developing economies (including 31 of the 47 Least Developed Countries). Data gaps and limited deployment in these countries typically lead to the use of standard assumptions^19^, which introduce inaccuracies into technoeconomic modelling^17^. The availability of cost of capital estimates from our modelling - with a strong temporal granularity and evaluated at both a technology and country level – addresses current data gaps and will ensure that researchers, policymakers and other energy stakeholders can use context-relevant information for ESMs and other technical evaluations. Avoiding the use of standard assumptions is especially important in developing economy contexts, which face much higher financing costs than advanced economies and so where the use of inaccurate assumptions would substantially impair the utility of technical insights.

**Fig. 3:**
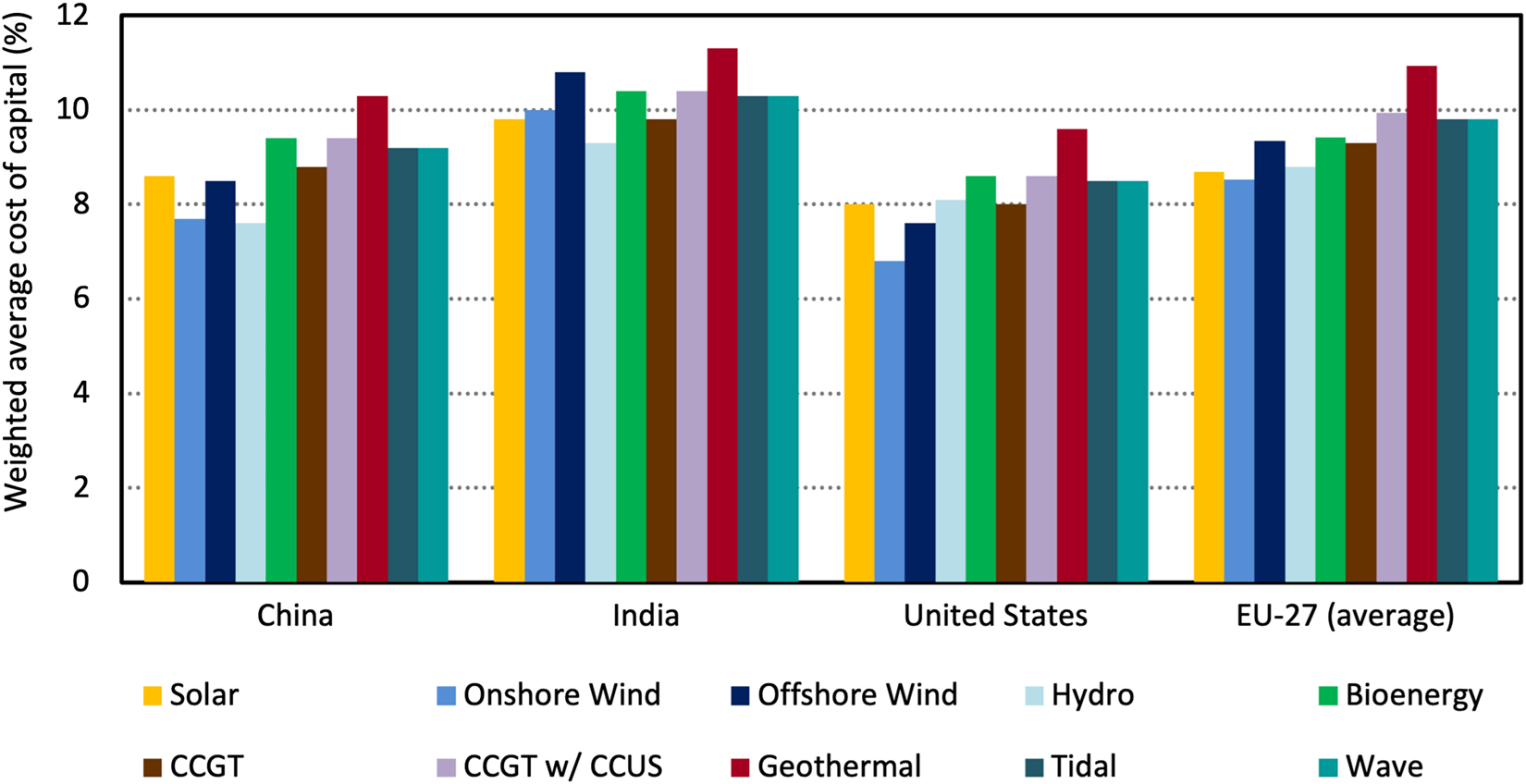
The range of estimated cost of capital for ten power generation technologies in 2024, plotted for India, China, the EU-27 (average) and the United States. Data is reported in nominal terms. These countries and regions were selected as they collectively account for the majority of global greenhouse gas emissions and current electricity consumption.

Our modelling approach also allows us to estimate the cost of capital at a technology granular level, covering 10 energy technologies selected due to their applicability to the energy transition across many countries. Figure 3 presents the range of estimates for a given year by technology, for the four largest contributing countries or economic blocs to global greenhouse gas emissions. These results highlight the importance of using technology-specific costs of capital, where possible, given the not insubstantial differences between technologies^35^ The capital intensity of some of the technologies presented here means that using standard assumptions across all technologies could bias their competitiveness (e.g., the internal evaluation of their levelised cost of electricity) in ESMs, introducing inaccuracies in modelled mitigation pathways.

**Fig. 4:**
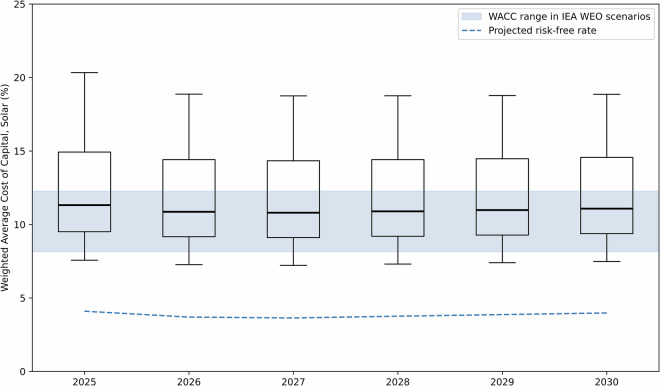
Projected costs of capital ranges for solar PV across the globe, benchmarked against the range taken in the IEA’s World Energy Outlook^45^. Data is reported in nominal terms, with the real costs of capital reported in the World Energy Outlook’s methodology report were converted to nominal using an assumed long-term inflation rate of 3%. Outliers from this work’s scenario predictions for solar PV are not included in the mapped boxplots. Projected risk-free rates shown are the U.S. 10 Year Treasury Bond scenario assumptions made by the Congressional Budget Office at the time of writing^56^.

Our model also provides temporally granular estimates for the cost of capital, covering a period of 15 years from 2015–2030 (including short term projections for the five years from 2025, see Methods). The horizon year of 2030 was selected due to the availability of data on projected changes in risk-free rates and national renewable targets for 2030. Projections beyond this point should be based on explicit modelling of technology evolution, policy development and capital market dynamics, due to larger uncertainties over how these parameters are likely to evolve in the medium- or long-term. Figure 4 presents the range of cost of capital for solar PV across the 176 countries evaluated, with the projected yearly risk-free rate also illustrated. It also provides a comparison against the assumed cost of capitals across regions modelled in the IEA’s World Energy Outlook 2024, which range between 4–7% for solar PV and onshore wind, 5–8% for offshore wind and 8–9% otherwise^45^.

## Methods

**Fig. 5:**
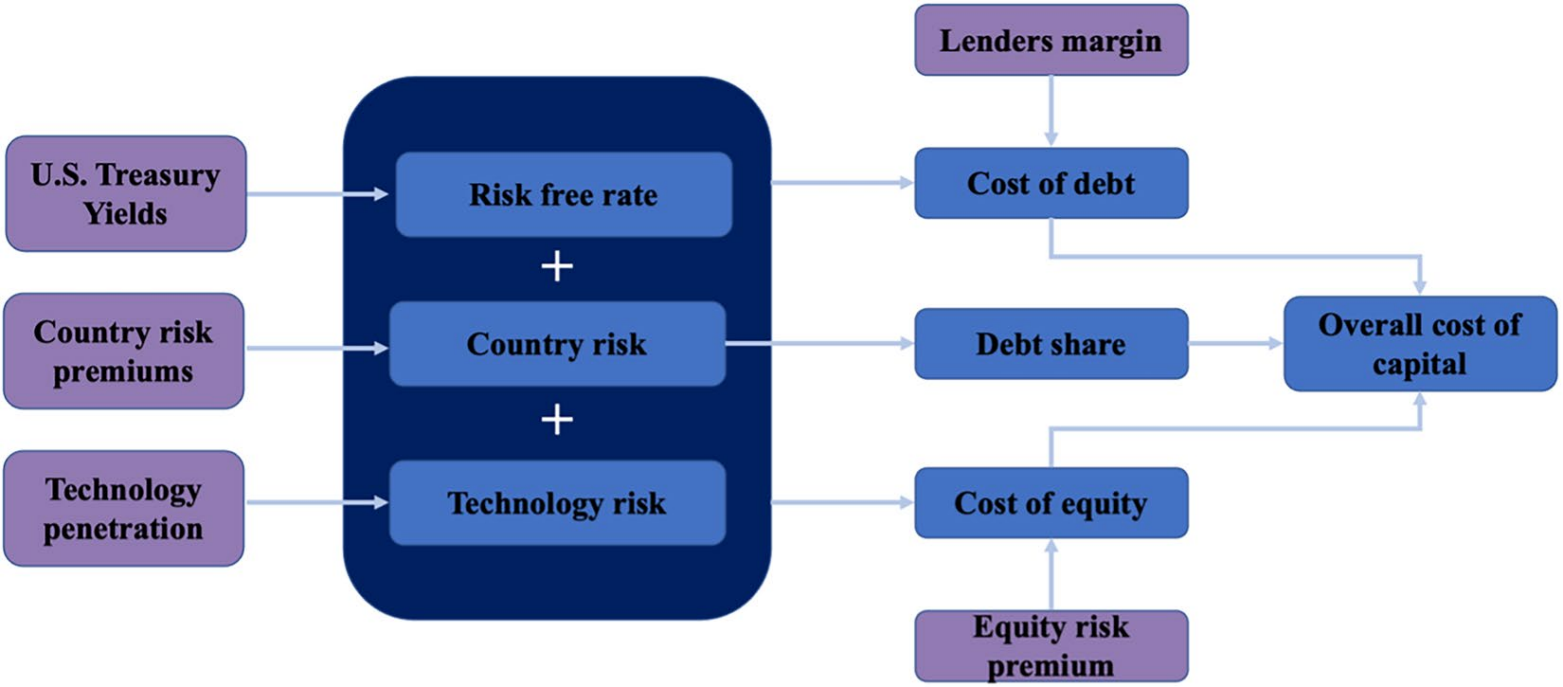
A schematic of the approach used in this work to calculate the cost of capital for a given year, country and technology combination. Contributions to the cost of debt and cost of equity from the risk-free rate, country level risk, technology risk and instrument risk are disaggregated and then combined with assumptions over the likely debt share for the given combination to arrive at the overall cost of capital. Inputs are highlighted in purple whilst intermediate and final parameters are in dark blue.

Figure 5 outlines the approach used to develop this dataset, which advances upon the benchmarking tool developed by IRENA^34^ and used in Calcaterra *et al*.^41^. It involves disaggregation of the cost of debt and cost of equity by assessing contributing risk factors at a country and technology level: the risk-free rate, the instrument premium, the country risk premium and a technology risk premium. The cost of capital is then evaluated by applying assumptions for the likely debt to equity split in each country for where the cost of debt and equity is estimated. All data in this dataset is reported in nominal terms and is calculated from the viewpoint of an international commercial financial institution (e.g., a large international bank lending at commercial rates), as a) domestic financing or public financing will differ in the risks they account for and b) international commercial investors will need to play a key role financing in the energy transition globally, particularly in developing countries^48^.

### Cost of Debt

Using Equation [1], the cost of debt (${C}_{D}^{c,t,g}$) was calculated for a given country (c), time period (t) and generation technology (g) through disaggregation into contributions from different categories of risk. The risk-free rate (${r}_{{rf}}^{c,t,g}$) was taken as the average yield on a 10-year U.S. Treasury Bond across all technologies, countries and time periods, a standard practice in financial analysis for international commercial investments. The debt lender’s margin (${r}_{{lm}}^{c,t,g}$) was taken as 2% in line with IRENA’s assumptions for large-scale infrastructure projects^34^, which was verified against AEW’s tracking of project finance loans for infrastructure investments (in which the average spread above reference rates is 1.91% for Europe and 2.29% for the rest of the world)^49^. Taking the lender’s margin as constant here assumes that lenders do not demand a higher margin for investments in emerging economies, which was a necessary assumption given limited data and that existing databases such as AEW are skewed towards OECD countries (and so may misrepresent lender behaviour). The country default spread (${r}_{{cds}}^{c,t,g}$) for each year was extracted from Damodaran’s annual estimates of the country risk premium^50^, which calculates country default spreads either based on sovereign bond credit ratings (if available) by S&P and Moody’s or spreads between the 10-year US Treasury bond and a sovereign bond of a similar duration denominated in euros or US dollars.

1 $${C}_{D}^{c,t,g}={r}_{{rf}}^{c,t,g}+{r}_{{lm}}^{c,t,g}+{r}_{{cds}}^{c,t,g}+{r}_{{tech}}^{c,t,g}$$

The technology premium (${r}_{{tech}}^{c,t,g}$) was evaluated based on the maturity of each technology in a given country, calculated using generation capacity data from Ember^51^. As in IRENA^34^, countries were categorised into Mature, Intermediate and Emerging markets for each technology, with technology-specific premiums and boundaries for market maturity applied based on technology maturity (see Table 1). The specific premiums and boundaries were based, as in IRENA^34^, on relationships between installed renewable capacity and risk premiums taken from PWC 2020^52^ and Egli 2020^53^. An additional 1% risk premium was added for offshore wind as compared to onshore wind based on maturity and scale considerations, in line with the additional risk premium reported by Europe Economics. Within the Intermediate maturity thresholds, a linear interpolation was applied to relate the change in market penetration to the risk premium, advancing on the approach followed by IRENA^34^ to avoid stepwise changes when technology penetration crosses a boundary. The authors also note that country-specific “technology risk premiums” encompass a wide range of market, project and company level risks that are challenging to capture in a single country average technology risk premium, so stakeholder engagement and additional risk evaluations to inform calibration of these values (e.g., addition of project-level risks) is key to the accurate depiction of costs of capital in ESMs.

**Table 1:** Breakdown of the market maturity boundaries and corresponding technology premiums for three key renewable technologies: solar photovoltaic, onshore wind, and offshore wind.

Technology	Mature		Intermediate		Emerging	
	Threshold (%)	Premium (%)	Threshold (%)	Premium (%)	Threshold (%)	Premium (%)
Solar PV	>10	1.5	10 > p > 5	3.25-0.35(p-5)	<5	3.25
Onshore Wind	>10	1.5	10 > p > 5	3.25-0.35(p-5)	<5	3.25
Offshore Wind	>10	2.5	10 > p > 5	4.25-0.35(p-5)	<5	4.25
Other technologies	>10	1.5 + r_tr_	10 > p > 5	3.25-0.35(p-3) + r_tr_	<5	3.25 + r_tr_

Technology premiums for other technologies reported by Europe Economics^39^ and included in this study were calculated by applying a relative premium (r_tr_), with corresponding grid penetration (p, on a capacity basis) for the given technology.

Technology coverage was extended from IRENA’s methodology to encompass 7 additional generation technologies (hydroelectric power, gas combined cycle turbines (CCGTs), biomass, CCUS-enabled CCGTs, geothermal, tidal and wave power, see Table 2), using technology-specific debt premium estimates from corporate bond ratings^39^. The same market maturity boundaries as solar PV and onshore wind were applied to these technologies, with the difference between debt premiums reported by Europe Economics used to calculate the new technology premium. As debt premium estimates are taken from a mature market (the United Kingdom), it should be noted that this approach might lead to conservative estimates of technology premiums in emerging and intermediate markets. To address these data gaps, future research should conduct similar in-depth risk analysis for emerging and intermediate markets.

**Table 2:** Breakdown of the relative technology premiums for the seven additional low-carbon technologies expanded using Europe Economics’ evaluation of the relative cost of debt for these technologies^39^.

Technology	Mature		Intermediate		Emerging	
	Relative premium (%)	Overall Premium (%)	Relative premium (%)	Overall Premium (%)	Relative premium (%)	Overall Premium (%)
Biomass	0.49	1.99	0.49	2.865	0.49	3.74
Gas power (CCGT)	−0.26	1.24	−0.26	2.115	−0.26	2.99
Gas power (CCUS)	0.49	1.99	0.49	2.865	0.49	3.74
Geothermal	1.7	3.2	1.7	4.075	1.7	4.95
Hydroelectric	0	1.5	0	2.375	0	3.25
Tidal	0.34	1.84	0.34	2.715	0.34	3.59
Wave	0.34	1.84	0.34	2.715	0.34	3.59

The relative premium is calculated from the difference in reported costs of debt between the given technology and solar PV, with the overall premium then calculated using the premiums for solar PV in Table 1.

### Cost of equity

Using Equation [2], the cost of equity (${C}_{E}^{c,t,g}$) was calculated for a given country (c), time period (t) and generation technology (g) by similarly disaggregating it into contributions from different categories of risk. Parameters are evaluated through the same approach used in Equation [1] for the cost of debt, with the equity risk premium (${r}_{{erp}}^{c,t,g}$) and country risk premium (${r}_{{country}}^{c,t,g}$) taken from Damodaran’s annual estimates of country risks and the global equity risk premium^50^, with country risk premiums based on sovereign bond premiums and country-level indexes of volatility. Equity risk premiums are provided at a global level and so are constant across technologies and countries, with country and technology-specific risks accounted through disaggregation of contributions into the four distinct categories in Equation [2].

2 $${C}_{E}^{c,t,g}={r}_{{rf}}^{c,t,g}+{r}_{{erp}}^{c,t,g}+{r}_{{country}}^{c,t,g}+{r}_{{tech}}^{c,t,g}$$

### Capital structure

A key factor influencing the debt-equity split for infrastructure projects is the level of perceived risk, with higher equity shares required for higher risk projects. The debt share is assumed to vary inversely with the country risk premium linearly between 40% and 80%, with the boundaries chosen based on the debt shares reported by the Diacore project^54^ (which typically ranged between 60–80% debt share but reached as low as 40% for projects fully exposed to market prices and so which carry some of the highest risks). It should be noted that these ranges were drawn from EU-27 markets which are more mature and regarded as lower risk compared to many developing countries, which underpinned the decision to extend the debt share range to 40%. Other parameters such as project size, financing structure and technology- or project-specific characteristics may also affect the capital structure, which were not considered here due to the national level scope of the estimation. Equation [3] outlines how the cost of capital (WACC), is calculated based on the tax rate (*τ*), debt share (*D*_*share*_), cost of debt (${C}_{D}^{c,t,g}$) and cost of equity (${C}_{E}^{c,t,g}$) for a given country (c), time period (t) and generation technology (g). The cost of debt and cost of equity were calculated using Equations [1] and [2]. Tax rates (*τ*) for each given year were taken from historical corporate tax rates reported by the Tax Foundation^55^, though this may bea conservative estimate as some countries have or do provide technology-specific corporate tax provision (which have not been included due to data availability issues).

3 $${{WACC}}^{c,t,g}={\left(1-{\tau }^{t}\right){{D}_{{share}}}^{c,t}C}_{D}^{c,t,g}+{\left(1-{{D}_{{share}}}^{c.t}\right)C}_{E}^{c,t,g}$$

### Projections to 2030

The cost of debt and cost of equity were projected forward to 2030 using estimates for how the corresponding variables in Equations [1, 2] are likely to evolve over the five years from 2025. Changes in the risk-free rate were based upon the U.S. Congressional Budget Office’s 5-year projections in yields on a 10-year U.S. Treasury Bond^56^. Debt margin was assumed to remain constant, as private infrastructure debt margins have remained roughly constant since 2008^57^. The equity risk premium for future years was assumed to be the same as the 2024 equity risk premium, which historically has only fluctuated slightly. Country risk premiums (${r}_{{country}}^{c,t+N,g}$) for N years’ time for a given country (c) and generation technology (g) were assumed to fall based on changes in GDP per capita using Equation [4]. Country default spreads were also projected forward using the same methodology, given that they both are closely linked to sovereign risk ratings which are strongly influenced by GDP per capita. Five-year projections of GDP per capita from 2024 were taken from the IMF’s World Economic Outlook^58^. The “learning rate” parameter (i) was taken as 0.2081, based on the examination of GDP per capita and country risk premiums in Sweerts *et al*.^46^.

4 $${r}_{{country}}^{c,t+N,g}={r}_{{country}}^{c,t,g}{\left(\frac{{{GDP}}^{c,t}}{{{GDP}}^{c,t+N}}\right)}^{i}$$

Technology risk estimates for future years used the same approach as for current and historic years, evaluated using projections of technology penetration by year. Grid penetrations were based on 2030 renewable targets from Ember’s 2030 Global Renewable Target Tracker^59^, which collates targets by technology and country for 96 countries and the European Union. Penetration was assumed to rise linearly in each country from 2025 to 2030. Country targets for generation technologies that were specified in terms of technology penetration were only available for 63 countries; where country targets were not available, technology penetration was held constant at 2024 levels. Countries for which 2030 targets were not available are principally developing countries, where a) country risk is typically the main driver of the cost of capital and b) where markets are largely "Immature" (as defined by this work based on grid penetration) for the majority of technologies examined in this work, so using the 2024 penetration does not influence the results substantively. Given that many energy system models use 2050 or beyond as a horizon for modelling – and so should adopt scenario-based approaches as proposed by Lonergan *et al*.^19^ for projections beyond 2030 – an area of value for future research would be in the development of standard scenarios e.g., linked to the Shared Socioeconomic Pathways (SSPs).

## Data Records

All data is recorded in Excel worksheets (csv format) and organised into both long and wide formats, which are accessible to the public at https://zenodo.org/records/17076925 (10.5281/zenodo.17076925^47^). Data is presented both in a wide format (with separate rows for each technology and country) and in a long format (with a value column and then indexer columns for the year, country, and technology) to maximise the ease of usability across a range of data analysis tools. ISO3 country codes are also included in both the wide and long formats to aid usability. Reported cost of capital values are given in the units of percentage points and given as nominal, post-tax terms in hard currency (as for many emerging market and developing economies loans are provided in hard currencies such as Euros or US Dollars, due to high inflation rates in local currencies).

## Technical Validation

**Fig. 6:**
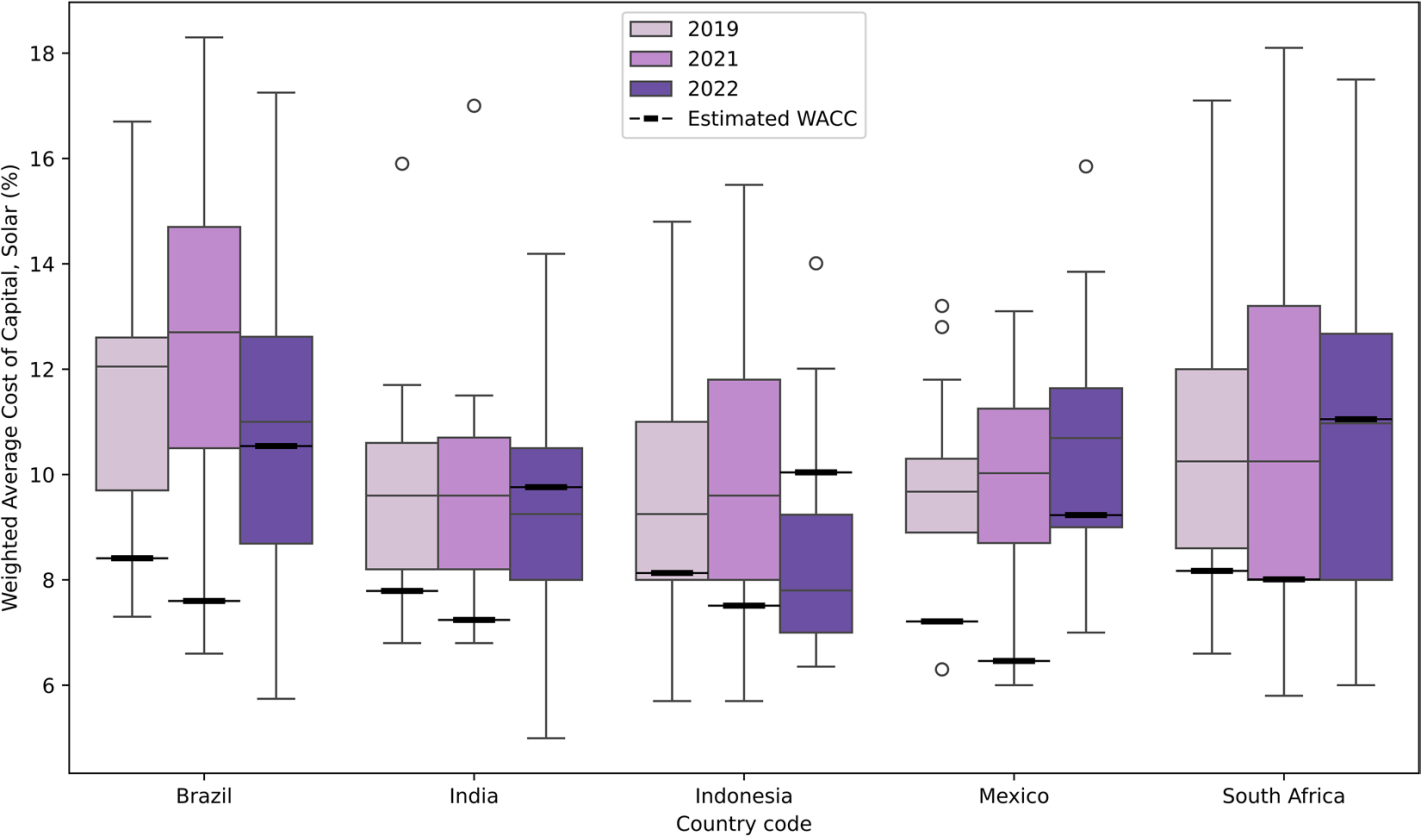
Benchmarking of the cost of capital estimates performed by this work against the project-level data on cost of capital in developing countries collected by the International Energy Agency’s Cost of Capital Observatory^23^. Estimates are highlighted in black for the corresponding years against the reported ranges in the Observatory, which gathers empirical project-level data from financiers and project developers. Circles outside the boxplots are used to highlight data outliers that are either 1.5x the corresponding interquartile range above the upper quartile or 1.5x it below the lower quartile, as is typical practice.

The methodological approach used here for cost of capital estimation, which extends on IRENA’s benchmarking tool, has been previously verified using expert surveys and interviews^34^ and peer reviewed in Calcaterra *et al*.^41^. Estimates produced here were also benchmarked and verified through comparisons with existing values in the literature, including those collated through the GNESTE database^60^, though (for reasons highlighted above) there is limited data available for developing countries. The largest source of project-level data is the International Energy Agency’s Cost of Capital Observatory, which collates financing term data for a select number of technologies in emerging and developing economies^23^. Figure 6 presents a comparison of the estimates from our model to the data collected by the IEA's Cost of Capital Observatory. As their data shows, the cost of capital can range substantially even for a given country, year and technology combination, due to the confluence of project, sponsor and financier-level risks. This work’ sestimates are within the range of data reported by the IEA and are typically in the lower two quartiles, which can be explained by a) the aim to create national cost of capital estimates, rather than project level estimates, which ensures a wide scope and broad applicability but does not include project-level risk premiums in scope and b) the inclusion of local currency financing in the IEA’s Cost of Capital Observatory, which can have much higher costs of capital than those from international markets denominated in hard currency. Given knowledge of the specific project and financier-level context, the effect of project-level risks (which can contribute up to 4% in additional risk premiums by one estimate from the literature^61^), currency denomination and/or de-risking programmes should then be added or subtracted to our national-level estimates. In the absence of project-level knowledge, the ranges displayed by the empirical data from the IEA’s Cost of Capital Observatory should be used to accurately account for the inherent uncertainty associated with calculating an average cost of capital value for a given year, country and technology combination. The use of data presented here should also not eschew sufficient treatment of uncertainty and sensitivity analyses.

Whilst the estimates and results presented here make substantial steps in addressing data gaps by providing historical and future estimates for ten power generation technologies across 176 countries, there are some limitations that must be considered when applying the data to energy systems modelling or other technoeconomic tools. The methodology has been constructed from the perspective of an international financier offering debt and equity financing at commercial rates, which may not depict the lower costs of financing available from local capital markets or domestic/public financiers (including financing at concessional rates). For the least developed countries - which are eligible for the highest concessional rates for infrastructure financing through the World Bank’s International Development Association - the data and methodology presented here may overestimate the cost of capital for key energy technologies. The methodology also does not account for sector-specific fiscal incentives that may provide energy developers with lower tax rates than national statutory corporate taxes and does not depict the influence of other fiscal and macroeconomic parameters on the capital structure of investments beyond country risk. Both of these factors may limit the project- or country-level accuracy of the cost of capital estimates presented here.

Currency mismatches between the investor and project cashflow also impact the overall cost of capital through currency depreciation and fluctuating exchange rates, which is not explicitly modelled here. International investors typically denominate project finance loans for infrastructure in “hard” currencies (e.g., USD, EUR) in developing countries, but there can often be a mixture of local- and hard-currency denominated investments in lower or upper middle-income markets. Whilst the cost of debt depicts subsequent currency dynamics and exchange risks through the country default spread (which is based on the difference in local vs hard currency denominated government bonds), it may not capture the effects of mixing local- and hard-currency denominated loans and/or the risk appetite of equity investors in respect to currency and exchange risks. Basing the risk-free rate on the U.S. Treasury yield, whilst a standard approach in the literature, may also introduce inaccuracies for the same reason. The approach here of modelling an international investor perspective with fully hard-currency denominated investments may therefore bias WACC estimates downwards (for markets with high local inflation and currency risk) or upwards (for markets where the local risk-free rate is lower than the U.S. Treasury yield).

International financiers also have their own, in-house models which are used to assess the potential default risks for a project and price the required returns accordingly, which typically includes more detailed cashflow analysis and statistical risk evaluations at the project level. The financial models used by investors are confidential^24,36^, making understanding and reflecting their approach for accounting for risks at different levels difficult. The methodology followed here is not designed to capture these project level risks and their interaction with country- and technology- level risks, which may over- or under-estimate the cost of capital (depending on project-level contextual factors). Investor treatment of risk may also differ markedly from the methodology used here to depict the influence of risk factors due to a range of behavioural biases^62^. Given the limitations discussed in the last few paragraphs and the high sensitivity of models to cost of capital assumptions, the data presented in this work should therefore not be used without sufficient sensitivity and uncertainty analysis.

## Usage Notes

Readers can initially download the datasets from 10.5281/zenodo.15227848. We also provide an interactive webtool that can be used to access and visualise the underlying data from the cost of capital estimation model that can be found at *wacc-forecaster.streamlit.app*. Data visualisation tools are a valuable way to engage audiences beyond technical specialists and so ensure that research is connected to the science-policy interface. Examples of successful platforms include the Demand.ninja web tool^63^ IEA’s Levelised Cost of Hydrogen Map^64^ and Ember Climate’s Electricity Data Explorer^65^.

An in-depth description of the possible usages of the webtool is beyond the scope of this article, but here we briefly summarise the potential usage of the constituent visualisation tools: (1) Map: visualises the cost of capital for the selected technology and year, providing data on the cost of debt, equity, tax rate and estimated debt share at a national level, (2) Global Estimates: allows for comparison of the cost of capital for a given technology and year and the contributions from different risk factors, (3) Country Projections: visualisation of the change in cost of capital over time for a given country and technology combination, (4) Technologies: visualises the range of cost of capital for selected technologies in a given country and (5) Calculator: allows for modification of the underlying assumptions to calculate a new cost of capital for a given year, country and technology.

## Data Availability

Data has been stored in a Zenodo repository, organised into both long and wide CSV formats, which is accessible to the public at https://zenodo.org/records/17076925 (10.5281/zenodo.17076925).

## Code availability

The scripts for data pre-processing and visualisation can be accessed in the GitHub repository, which can be found here: https://github.com/LukeHatton21/wacc-estimator.
